# Calcium-binding protein S100A14 induces differentiation and suppresses metastasis in gastric cancer

**DOI:** 10.1038/cddis.2017.297

**Published:** 2017-07-20

**Authors:** Min Zhu, Hongyi Wang, Jiantao Cui, Wenmei Li, Guo An, Yuanming Pan, Qingying Zhang, Rui Xing, Youyong Lu

**Affiliations:** 1 Key Laboratory of Carcinogenesis and Translational Research (Ministry of Education), Laboratory of Molecular Oncology, Peking University Cancer Hospital and Institute, Beijing 100142, China; 2 Key Laboratory of Carcinogenesis and Translational Research (Ministry of Education), Department of Surgery, Peking University Cancer Hospital and Institute, Beijing 100142, China; 3 Key Laboratory of Carcinogenesis and Translational Research (Ministry of Education), Department of Cell Biology, Peking University Cancer Hospital and Institute, Beijing 100142, China; 4 Department of Preventive Medicine, Guangdong Provincial Key Laboratory of Infectious Diseases and Molecular Immunopathology, Shantou University Medical College, Shantou 515041, China

## Abstract

S100A14 is a calcium-binding protein involved in cell proliferation and differentiation as well as the metastasis of human tumors. In this study, we characterized the regulation of S100A14 expression between biological signatures and clinical pathological features in gastric cancer (GC). Our data demonstrated that S100A14 induced the differentiation of GC by upregulating the expression of E-cadherin and PGII. Moreover, S100A14 expression negatively correlated with cell migration and invasion in *in vitro* and *in vivo* experimental models. Interestingly, S100A14 blocked the store-operated Ca^2+^ influx by suppressing Orai1 and STIM1 expression, leading to FAK expression activation, focal adhesion assembly and MMP downregulation. Taken together, our results indicate that S100A14 may have a role in the induction of differentiation and inhibition of cell metastasis in GC.

Gastric cancer (GC) is the third most important cause of global cancer mortality.^1^ Although improved treatment, such as surgery and chemotherapy, has been effective in reducing mortality, the 5-year survival rate of GC patients remain relatively low.^2^ Increasing studies have reported that metastasis is responsible for GC-related deaths by the dysregulation of multiple genes, including p53, c-met and k-ras.^3^ However, the mechanisms of cell differentiation, proliferation and metastasis remain largely unknown. Hence, searching for pathological diagnosis and metastasis-related biomarkers is necessary for the clinical prediction and assessment of GC.

The S100 protein family has been reported to contribute to multiple biological processes, such as growth, cell motility, signal transduction, transcription, cell survival and apoptosis, which are related to normal development and tumorigenesis.^4^ Accumulating evidence has indicated that the dysregulation of S100 family members correlates with tumor progression in various types of cancers, including breast cancer, liver cancer and colorectal cancer.^5, 6, 7, 8^ Specifically, S100A2,^9^ S100A4^10^ and S100A6^11^ are associated with tumor differentiation and promoted tumor growth. In addition, S100A4,^10, 11, 12, 13^ S100A8/A9,^14^ S100P^15^ and S100A13^16^ have been shown to be involved in tumor invasion and metastasis. In our previous study, we explored and identified a panel of differentially expressed genes between intestinal type and diffuse type GC, including genes encoding S100 protein family members, by gene microarray and experimental studies of GC.^17^ We further identified the varied expression of seven S100 members in GC tissues and cell lines, including S100A2, S100A6, S100A10, S100A11, S100A14, S100P and S100B, based on our previous microarray screening.^18^ Interestingly, the effect of S100A14 expression on tumor behavior and progression was controversial in different tumors, and its role in GC has not yet been clarified.

Our previous work showed that decreased expression of S100A14 was associated with poor prognosis in GC.^18^ Hence, we will illustrate the previously unknown tumor-related effect of S100A14 on tumor differentiation, cell proliferation and metastasis in GC.

## Results

### Decreased expression of S100A14 is positively associated with poor differentiation and poor prognosis in GC

**Figure 1:**
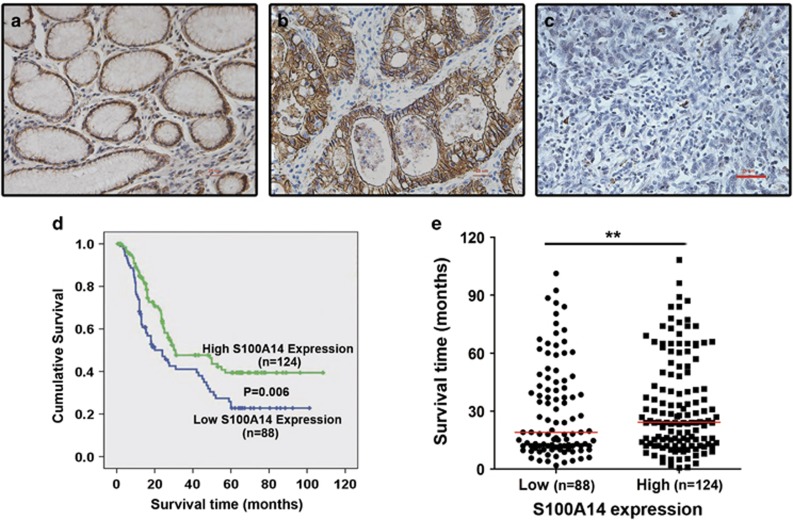
Decreased S100A14 expression was associated with poor survival in GC. (**a**–**c**) Representative images of S100A14 expression in normal gastric mucosa tissue (**a**), intestinal type GC tissue (**b**) and diffuse type GC tissue (**c**). Scale bar, 25 *μ*m. (**d**) Kaplan–Meier analysis of overall survival in patients with variable S100A14 expression. (**e**) Scatter diagram showed that patients with lower S100A14 expression displayed shorter overall survival after surgery. **P<0.01

To clarify the clinical significance of S100A14, we first used immunohistochemistry to screen the expression of S100A14 in 485 cases of primary GC tissues and 289 cases with matched adjacent normal tissues by immunohistochemistry. Our results confirm that there was no significant difference in S100A14 expression between normal tissues (Figure 1a) and tumor tissues (*P*=0.313, Supplementary Table S2). Intriguingly, 61.3% (184/300) of intestinal type GC (well-differentiated tumors) was identified with high S100A14 expression (Figure 1b), but only 39.2% (38/97) of diffuse type GC (poorly differentiated tumors) expressed the S100A14 protein (Figure 1c).

Correlation study results showed that S100A14 expression was positively correlated with Lauren classification (*P*<0.001) and differentiation (*P*=0.044), but negatively correlated with tumor depth (*P*=0.014), lymph node status (*P*=0.011) and distant metastasis (*P*=0.010; Table 1). Kaplan–Meier survival analysis showed that GC patients with low S100A14 expression had a worse outcome than those with high S100A14 expression (*P*=0.006, Figure 1d). The median survival period for patients with low S100A14 expression (40.1 months; *n*=88) was significantly shorter than that for patients with high S100A14 expression (56.5 months; *n*=124) (Figure 1e). These results imply that S100A14 might have a role in regulating tumor differentiation and inhibiting cell migration and invasion.

### S100A14 regulates GC cell differentiation by upregulating the expression of PGII and E-cadherin

**Figure 2:**
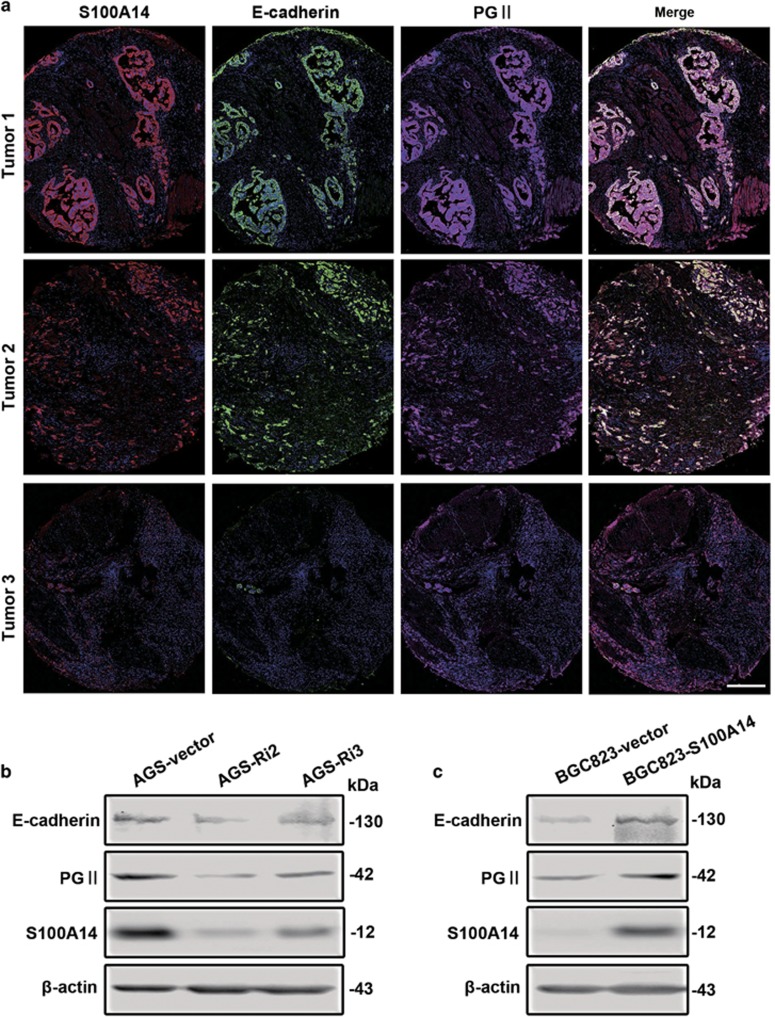
Functional effects of S100A14 expression on differentiation in GC cells. (**a**) Representative multiple fluorophore-antibody (blue: nucleus; red: S100A14; green: E-cadherin; magenta: PGII) staining on the same tissue samples. Scale bar, 50 *μ*m. (**b** and **c**) Western blot analysis data show that interference of S100A14 expression downregulated the expression of E-cadherin and PGII in AGS cells (**b**), and S100A14 overexpression upregulated the expression of E-cadherin and PGII in BGC823 cells (**c**)

To understand the mechanism whereby S100A14 promotes the differentiation of GC, we determined the expression of E-cadherin and PGII, which are gastric mucosa differentiation and mature markers, under altered S100A14 expression levels. We observed that the localization of S100A14 and E-cadherin protein expression was overlapped on the cell membrane in most well-differentiated GC tissues (Figure 2a) and that S100A14 and PGII co-localized in the same tissues. Statistical analysis indicated that there was also a statistically correlated trend between the positive expression of S100A14 and E-cadherin (*r*=0.981, *P*<0.001; Figure 2a). A similar correlation was found between S100A14 and PGII(*r*=0.885, *P*<0.001; Figure 2a). These observations indicate that the S100A14 protein can serve as a potential marker for tumor differentiation in GC.

In addition, we established S100A14 downregulated stable transfectants in AGS cells and S100A14 overexpressed stable transfectants in BGC823 cells (Supplementary Figure 1). Intriguingly, we observed that E-cadherin and PGII were downregulated when the expression of S100A14 was inhibited in AGS cells (Figure 2b) and that the overexpression of S100A14 increased the expression of E-cadherin and PGII in BGC823 cells (Figure 2c). These results suggest that S100A14 is an important mediator of differentiation in GC.

### S100A14 inhibites GC cell migration and invasion *in vitro* and *in vivo*

**Figure 3:**
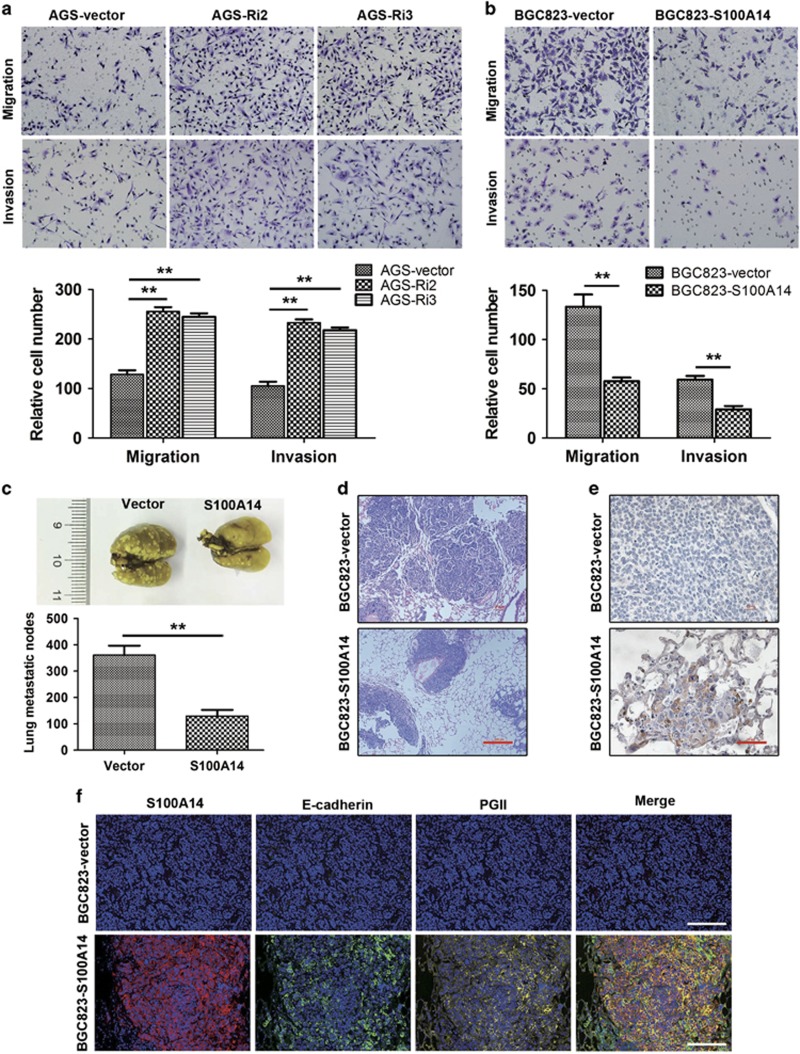
Functional effects of S100A14 on cell migration and invasion. (**a** and **b**) Cell migration and invasion were performed with the transwell assay. S100A14 knockdown increased cell migration and invasion in AGS cells (**a**), and S100A14 overexpression decreased cell migration and invasion in BGC823 cells (**b**). Quantitative analysis of the cells that migrated across the matrix membrane (lower). (**c**) Representative image of lung metastasis. Cell lines stably expressing S100A14 and vector control were injected into mice via the tail vein, and lungs were isolated from mice and photographed 6 weeks later. The data are shown as the mean±S.D. from five pulmonary lobes in five mice. (**d**) H&E staining for tumors in mice lungs. Scale bars, 100 *μ*m. (**e**) IHC staining shows the S100A14 expression status in lung tissues. Scale bars, 25 *μ*m. (**f**) Representative multiple fluorophore-antibody (blue: nucleus; red: S100A14; green: E-cadherin; yellow: PGII) staining in the same metastatic nodes. Scale bars, 50 *μ*m. The data are shown as the mean±S.D. from three separate experiments. **P<0.01

We next investigated whether S100A14 has an important role in GC cell migration and invasion. As shown in Figure 3a, the number of AGS-Ri2 and AGS-Ri3 cells that invaded the matrix membrane or matrigel membrane was dramatically increased compared with that of AGS-vector cells. In contrast, the number of BGC823-S100A14 cells that invaded the matrix membrane or matrigel membrane was dramatically decreased compared with that of BGC823-vector cells (Figure 3b).

Moreover, we used a mouse multiple pulmonary carcinoma metastasis model to further investigate the role of S100A14 in inhibiting GC metastasis. Notably, compared with BGC823-vector cells, the total cell foci of BGC823-S100A14 that metastasized to the lungs was reduced by 64.0% (*P*<0.01; Figure 3c) as shown by H&E staining (Figure 3d). The expression of S100A14 in BGC823-S100A14 xenografts and BGC823-vector xenografts was confirmed by immunohistochemistry (Figure 3e), and the expression of E-cadherin and PGII also confirmed by histo-cytometry in metastatic nodes (Figure 3f). These results indicated that overexpression of S100A14 induced differentiation and suppressed the metastasis of BGC823 in NOD/SCID mice.

### S100A14 inhibites GC cell migration and invasion by modulating the store-operated Ca^2+^ influx

**Figure 4:**
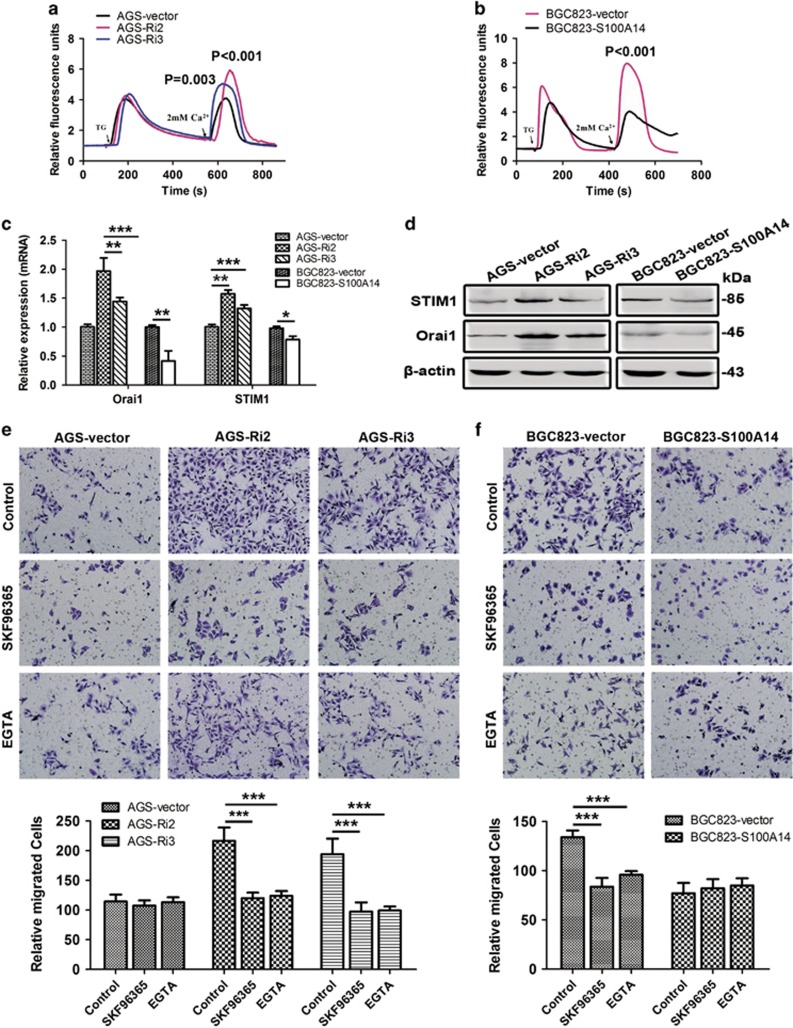
S100A14 blocked the store-operated Ca^2+^ influx and inhibited GC cell migration by affecting the expression of Orai1 and STIM1. (**a** and **b**) S100A14 knockdown increased the store-operated Ca^2+^ influx in AGS cells (**a**), and S100A14 overexpression decreased the store-operated Ca^2+^ influx in BGC823 cells (**b**). TG, thapsigargin, 4 mM. The fluorescence values of cells at rest time as references, and the relative fluorescence values are presented as the mean±S.D. of 50 random cells. (**c** and **d**) Real-time PCR and western blot analyses of the mRNA and protein levels of Orai1 and STIM1, respectively. S100A14 knockdown increased the expression of Orai1 and STIM1 in AGS cells (**c**). Conversely, S100A14 overexpression decreased Orai1 and STIM1 expression in BGC823 cells (**d**). (**e** and **f**) SKF96365 (20 mM) and EGTA (2 mM) blocked the function of S100A14 in inhibiting migration in AGS cells (**e**) and BGC823 cells (**f**). The data are shown as the mean±S.D. of three experiments. *P<0.05; **P<0.01; ***P<0.001

S100 proteins exert their function depend on calcium and has been implicated in the regulation of Ca^2+^ homeostasis. To understand the underlying mechanisms involved in the suppression effects of S100A14 on metastasis in GC, we first investigated the effect of S100A14 on the intracellular calcium levels. As shown in Figure 4a, the influx of store-operated Ca^2+^ was increased in AGS-Ri2 and AGS-Ri3 cells compared with AGS-vector cells. Consistent with this finding, S100A14 overexpression in BGC823 cells blocked the store-operated Ca^2+^ influx (Figure 4b).

ORAI and STIM proteins have been reported to be responsible for store-operated Ca^2+^ influx. Interestingly, the real-time PCR and WB results showed that the expression of Orai1 and STIM1 was significantly upregulated in AGS-Ri2 and AGS-Ri3 cells compared with that in AGS-vector cells (Figures 4c and d). Consistent with this finding, the expression of Orai1 and STIM1 was significantly downregulated in BGC823-S100A14 cells compared with that in BGC823-vector cells (Figures 4c and d). These data indicated that S100A14 might block the store-operated Ca^2+^ entry by downregulating Orai1 and STIM1.

In addition, SKF96365 (an inhibitor of store-operated Ca^2+^ entry, Abcam) could significantly inhibit AGS-Ri2 and AGS-Ri3 cell migration and invasion induced by S100A14 deficiency (Figure 4e and Supplementary Figure 2). Similar results were observed in AGS-Ri2 and AGS-Ri3 cells treated with EGTA: cell migration and invasion were decreased, while extracellular Ca^2+^ was chelated (Figure 4e and Supplementary Figure 2). Similarly, in BGC823-vector cells, SKF96365 and EGTA treatments decreased cell migration and invasion (Figure 4f and Supplementary Figure 2). Hence, these data demonstrated that S100A14 inhibited gastric cancer cell migration and invasion by modulating the store-operated Ca^2+^ influx.

### S100A14 regulates focal adhesion turnover through modulating the store-operated Ca^2+^ influx

**Figure 5:**
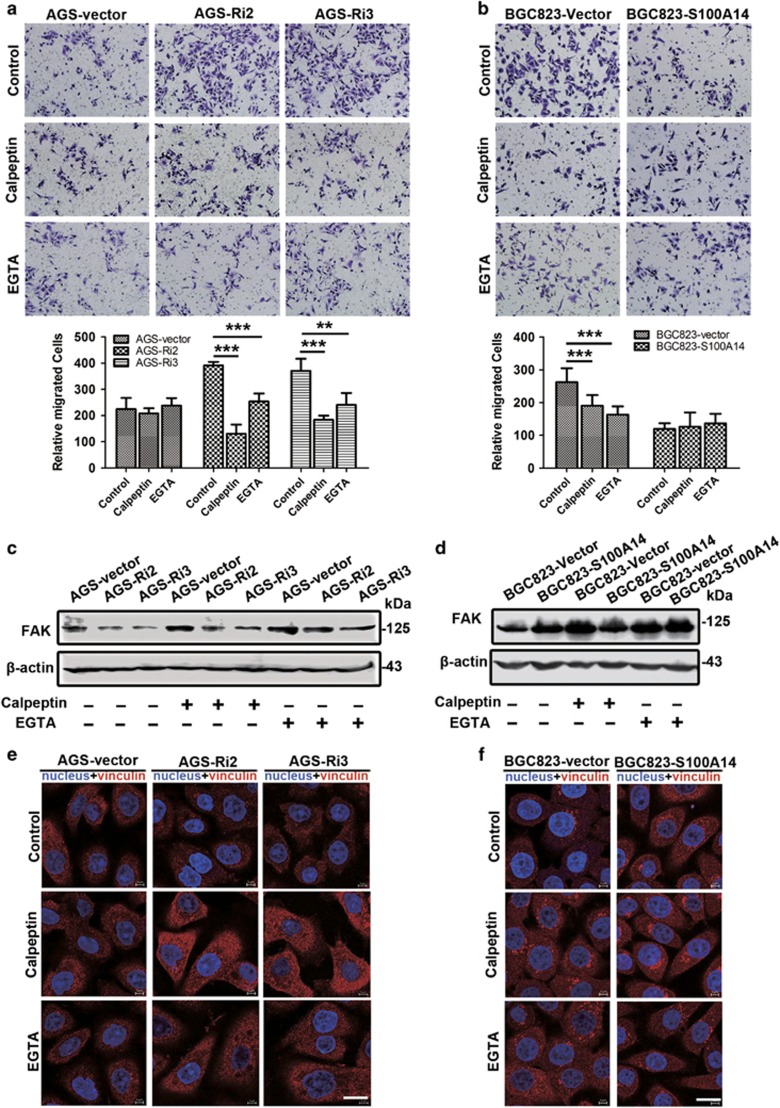
S100A14 inhibited cell migration through modulating focal adhesion. (**a** and **b**) Calpeptin (20 *μ*M) and EGTA (2 mM) decreased S100A14 knockdown-induced migration of AGS cells (**a**) and the migration of S100A14-deficient BGC823 cells (**b**). Quantitative analysis of the cells that migrated across the matrix membrane (lower). The data are shown as the mean±S.D. of three experiments, **P<0.01, ***P<0.001. (**c**) Western blot analysis showed that S100A14 knockdown induced the downregulation of FAK, and calpeptin and EGTA abrogated the decrease in FAK expression induced by S100A14 deficiency. (**d**) S100A14 overexpression upregulated FAK expression, and calpeptin and EGTA also upregulated FAK expression in BGC823. (**e** and **f**) Immunofluorescence analysis of vinculin expression in AGS and BGC823 cells. S100A14 knockdown decreased focal adhesion disassembly in AGS cells, and calpeptin and EGTA rescued S100A14 knockdown-induced focal adhesion disassembly (**e**); whereas S100A14 overexpression increased focal adhesion disassembly in BGC823 cells, and calpeptin and EGTA decreased the focal adhesion disassembly in S100A14-deficient BGC823 cells (**f**). Red indicates vinculin staining. Blue indicates nuclear staining. Scale bar, 5 *μ*m

It is known that increasing cellular Ca^2+^ levels could promote the activity of the tyrosine kinase FAK (focal adhesion kinase) and the calcium-dependent protease calpain in focal adhesions.^19^ Transwell assays showed that calpeptin (the inhibitor of calpain, Abcam) and EGTA decreased the migration and invasion induced by interfering with S100A14 expression in AGS cells (Figure 5a and Supplementary Figure 2) and S100A14-deficient BGC823 cells (Figure 5b and Supplementary Figure 2). WB results showed that the expression of FAK was significantly decreased in AGS-Ri2 and AGS-Ri3 cells compared with that in AGS-vector cells (Figure 5c), whereas FAK expression was increased in BGC823-S100A14 cells compared with that in BGC823-vector cells (Figure 5d). FAK expression was rescued after the AGS-Ri2 and AGS-Ri3 cells were treated with calpeptin (Figure 5c). We also chelated extracellular Ca^2+^ with EGTA, and then FAK expression was also rescued (Figure 5c). Similar results observed in BGC823-vector cells were consistent with that observed in AGS-Ri2 and AGS-Ri3 cells (Figure 5d). These results implied that S100A14 expression could induce FAK protein destabilization, leading to the inhibition of GC cell metastasis.

Furthermore, we observed the alteration of focal adhesions by immunostaining for vinculin, which has an important role in cell migration and invasion. As shown in Figure 5e, vinculin staining showed that the numbers of small punctate patterns of focal adhesions in AGS-Ri2 and AGS-Ri3 cells were less than those in AGS-vector cells. In addition, compared with BGC823-vector cells, BGC823-S100A14 showed more punctate patterns of small focal adhesions (Figure 5f). S100A14 deficiency reduced the small focal adhesions, and these focal adhesions were partly rescued by calpeptin and EGTA (Figures 5e and f). These results indicated that S100A14 inhibited cell migration and invasion by impairing focal adhesion, which is modulated by the store-operated Ca^2+^ influx.

### S100A14 downregulates MMP expression by modulating the store-operated Ca^2+^ influx

**Figure 6:**
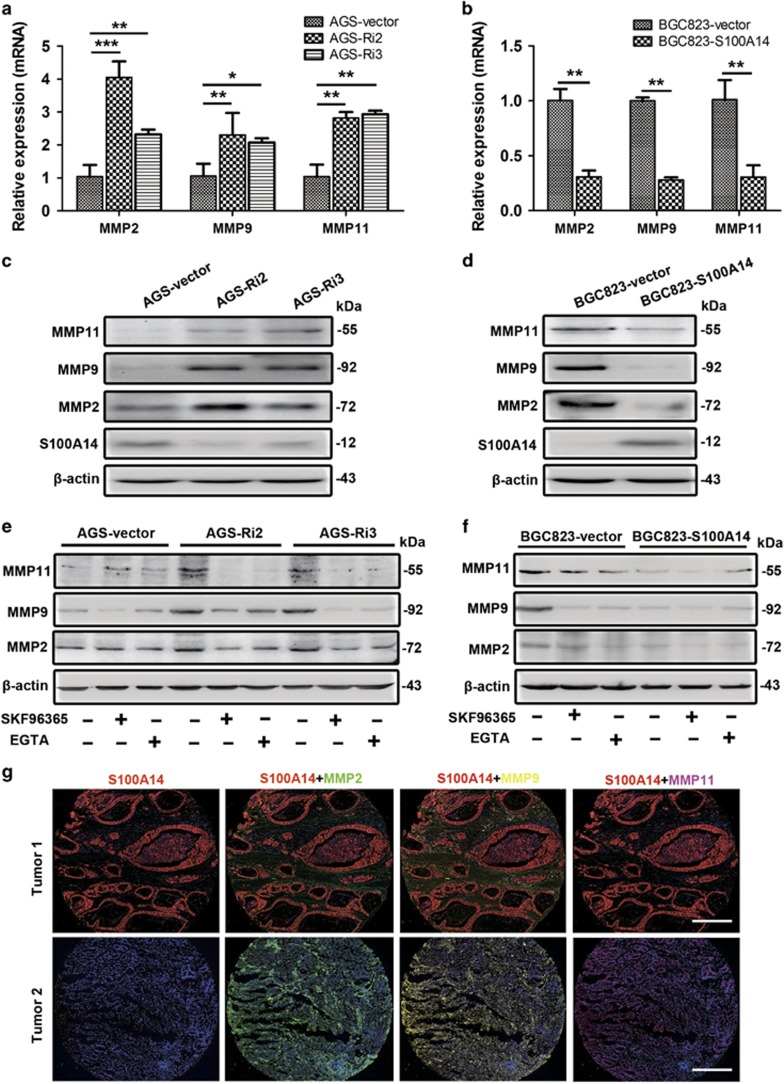
S100A14 inhibited cell invasion through downregulating MMPs expression. (**a**–**d**) Real-time PCR and western blot analyses of MMP2, MMP9 and MMP11 expression. S100A14 knockdown increased the mRNA and protein levels of MMP2, MMP9 and MMP11 in AGS cells (**a** and **c**). S100A14 overexpression decreased their expression in BGC823 cells (**b** and **d**). (**e** and **f**) Western blot analyses of MMP2, MMP9 and MMP11 expression. SKF96365 (20 mM) and EGTA (2 mM) reversed the upregulation of MMP2, MMP9 and MMP11 in S100A14 knockdown AGS cells (**e**). And SKF96365 and EGTA decreased the expression of MMPs in S100A14-deficient BGC823 cells (**f**). (**g**) Representative multiple fluorophore-antibody staining for S100A14 (red), MMP2 (green), MMP9 (yellow) and MMP11 (magenta) on the same tissue samples. DAPI-stained nuclei (blue). Scale bar, 50 *μ*m. The data are shown as the mean±S.D. of three experiments. *P<0.05; **P<0.01; ***P<0.001

MMPs are also important in regulating cell migration and invasion through digesting the extracellular matrix, which interacts with focal adhesions.^20^ Real-time PCR and WB results showed that the expression of MMP2, MMP9 and MMP11 was significantly upregulated in AGS-Ri2 and AGS-Ri3 cells compared with that in AGS-vector cells (Figures 6a and c). In addition, their effect could be rescued by pretreatment with SKF96365 and EGTA (Figure 6e). Similarly, overexpression of S100A14 in BGC823 increased the mRNA and protein levels of MMP2, MMP9 and MMP11 (Figures 6b and d), and SKF96365 or EGTA pretreatment decreased the expression of MMPs in S100A14-deficient BGC823 cells (Figure 6f). These data suggested that S100A14 reduced the expression of MMPs, which was dependent on blocking the Ca^2+^ influx.

By histo-cytometry, we further analyzed the correlation between S100A14 and MMPs in 50 GC tissues. Statistical analysis indicated that S100A14 protein expression was significantly inversely correlated with MMP2 (*r*=−0.665; *P*<0.001), MMP9 (*r*=−0.501; *P*<0.001) and MMP11 (*r*=−0.724; *P*<0.001) expression (Figure 6g).

## Discussion

Tumor cell differentiation strongly correlates with a malignant phenotype.^21, 22^ S100 proteins are involved in these complex processes; in particular, the variation in and role of S100A14 in GC are unclear. In this study, we found that decreased S100A14 expression was associated with poor differentiation, tumor depth, lymph node status and metastasis. Specifically, our findings demonstrate that S100A14 induced GC differentiation and suppressed metastasis, leading to better prognosis for GC patients. These results suggest that S100A14 could serve as a novel differentiation marker for predicting the clinical outcome for GC.

As S100A14 was identified from lung cancer in 2002,^23^ it has been shown to have an important role in cell growth,^24, 25, 26^ differentiation^27, 28^ and metastasis^24, 25, 29, 30, 31^ in many types of tumors. Our previous study^18^ and this study revealed that increased S100A14 expression was detected in well-differentiated tumors and associated with better outcomes for GC patients. However, the molecular mechanism of S100A14 involvement in GC cell differentiation and cancer metastasis remains poorly understood. S100A14 was demonstrated to be transcriptionally regulated by JunB and involved in ESCC cell differentiation through upregulating involucrin (IVL) and filaggrin (FLG) expression.^28^ In our study, S100A14 significantly correlated with the differentiation of gastric mucosa and the statuses of the mature markers PGII^32, 33^ and E-cadherin^34^ in GC tissue, and PGII and E-cadherin expression was altered with S100A14 expression in GC cells. Another previous study showed that S100A14 expression resulted in G1-phase cell cycle arrest and inhibited cell growth but failed to induce cell apoptosis during the process of promoting the terminal differentiation of ESCC cells.^28^ However, our cell growth assay and animal model data showed that S100A14 had no effect on GC cell growth *in vitro* and *in vivo* (Supplementary Figure 3), which is consistent with the clinical feature, namely, the lack of a significant difference in S100A14 expression between normal tissues and tumor tissues. This result suggests that S100A14 modulates differentiation but may not have an important role in tumor proliferation in GC. Notably, the role of S100A14 in GC cell proliferation was consistent with the findings of another study suggesting that S100A14 had no significant effect on cell growth in esophageal cancer.^29^

The effect of S100A14 on tumor metastasis remains controversial. Elevated S100A14 promotes the metastasis of tumor cells and induces worse survival in breast cancer,^35, 36^ ovarian tumors^24^ and hepatocellular carcinoma.^25^ However, S100A14 inhibits the invasive potential of oral squamous cell carcinoma^31^ and urothelial carcinoma,^30^ and S100A14 expression is inversely associated with multiple lymph node metastases of small intestinal adenocarcinomas^37^ and distant metastasis of colon cancer.^27^ S100A14 may have different roles in various kinds of tumors and depend on different potential signaling pathways. S100A14 was reported to be either an inducer or an inhibitor of cell invasion dependent on p53 status.^29^ Our study is the first to discover that S100A14 has an important role in suppressing GC cell migration and invasion through blocking the Ca^2+^ influx.

It is known that the interaction of S100 with other proteins is dependent on binding with Ca^2+^, and interactions such as S100P-ezrin^38^ and S100A4-Smad3^39^ have been identified to be dependent on Ca^2+^ and to promote cell metastasis. These S100 proteins always exert their functions through cytoplasmic calcium-dependent mechanisms. In contrast to other S100 genes, the calcium-binding site of S100A14 is mutated, leading to the failed binding between S100A14 and calcium,^40^ which suggests that some potential calcium-associated pathways that do not require direct binding with calcium should be further investigated. Interestingly, in our study, S100A14 blocked the store-operated Ca^2+^ influx, and cells with S100A14 expression decreased spontaneously calcium fluorescence at rest, in contrast to other cells that do not express S100A14. Although S100A14 has a low affinity for calcium, S100A14 may indirectly affect Ca^2+^ signaling. Unlike a previous study that showed that S100A10 could bind to TRPV5 or TRPV6 to affect Ca^2+^ channel activity,^41^ we found that S100A14 could block the Ca^2+^ influx by downregulating Orai1 and STIM1 expression, leading to relatively low intracellular calcium levels. Ca^2+^ is known to have an important role in cellular migration, invasion and motility via the regulation of various kinases,^42^ including calpain, which results in the proteolysis of E-cadherin.^43^ Our findings demonstrate that S100A14 may inactivate calpain through blocking the Ca^2+^ influx, resulting in the upregulation of E-cadherin, which serves as a differentiation marker and prevents GC metastasis.^33, 44^ In accordance with the function of LKB1,^21^ GATA-3^22^ and RARRES3,^45^ S100A14 inhibits tumor metastasis by regulating differentiation and adhesion in GC.

Matrix metalloproteinases (MMPs) have a vital role in the tumor invasion process by degrading multiple elements of the extracellular matrix (ECM).^20, 46^ The 100A14 protein suppressed OSCC cell invasion by downregulating the expression of MMP1 and MMP9,^31^ and S100A14 either promoted or inhibited cell invasion by regulating MMP2 in a p53-dependent manner.^29^ Consistent with previous reports,^47, 48, 49^ our findings imply that S100A14 not only inactivates calpain and stabilized focal adhesion kinase (FAK) but also downregulates the expression of MMPs via decreasing cellular Ca^2+^ levels. Therefore, the molecular mechanism by which the Ca^2+^ signal induced by the loss of S100A14 affects the expression of MMPs should be further studied in the future.

**Figure 7:**
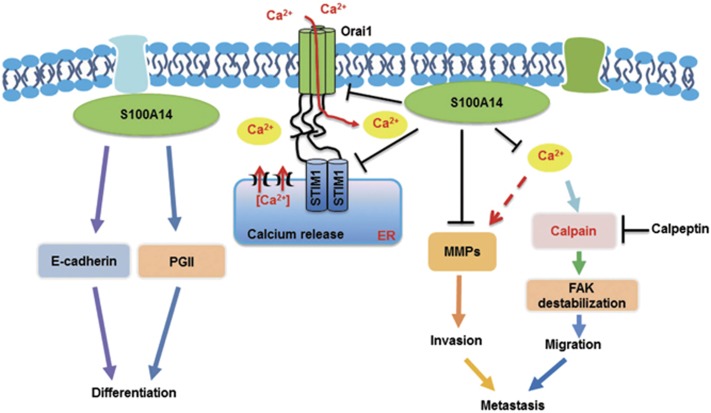
Schematic of S100A14-mediated effects on the differentiation and metastasis of GC cells. S100A14 induced GC cell differentiation through upregulating PGII and E-cadherin expression. In addition, S100A14 inhibited GC cell migration and invasion by blocking the store-operated Ca^2+^ influx and decreasing MMP expression in GC cells

Collectively, our results indicate that decreased S100A14 expression is associated with poor differentiation, distant metastasis and poor prognosis in GC. Our study found that S100A14 regulates tumor cell differentiation by altering PGII and E-cadherin expression, and S100A14 expression is significantly correlated with PGII and E-cadherin expression in GC tissue. Furthermore, S100A14 has an important role in inhibiting the migration and invasion of gastric cancer cells by blocking the store-operated Ca^2+^ influx, leading to FAK activation and decreased MMP expression in GC cells (Figure 7). Our findings demonstrate that S100A14 is a potential biomarker for predicting tumor metastasis and prognosis in gastric cancer.

## Materials and methods

### Tissue specimens

The tissue specimens in this study were scrutinized and approved by the Ethics Committee of Peking University Cancer Hospital. The formalin-fixed and paraffin-embedded tissues for immunohistochemistry (IHC) were used to construct a tissue microarray. In total, tissue samples from 440 cases of primary gastric tumor and 289 cases with matched normal tissues were obtained from Peking University Cancer Hospital, and 212 cases of GC had follow-up data. All tissue specimens were from the patients without radiation therapy and chemotherapy.

### Immunohistochemistry

Immunohistochemistry was performed using a standard technique with the avidin-biotinylated peroxidase complex as described previously.^18^ The slides were incubated with polyclonal anti-S100A14 antibody (1:100, Proteintech Group, Chicago, IL, USA) at 4 °C overnight. Diaminobenzidine (DAKO, Carpinterin, CA, USA) staining was used for detecting immunoreactivity. The intensity of immunoreactivity was graded 0, 1+, 2+ and 3+ for no staining, weak, medium and strong staining, respectively. Scores of 0 and 1+ were regarded as low expression; scores of 2+ and 3+ were considered high expression.

### Histo-cytometry

Slides loaded with gastric cancer tissue microarrays were deparaffinized in xylene and rehydrated in a graded alcohol series. Antigen retrieval was performed in citrate buffer (pH 6.0) at 95 °C for 20 min and cooled down at room temperature. After the quenching of endogenous peroxidase in 3% H_2_O_2_, the slides were incubated with blocking reagent (ZSGB-BIO, Beijing, China) for 30 min at room temperature. Antigens were then successively detected using the Opal protocol: each primary antibody was incubated at 4 °C overnight, followed by detection using HRP-conjugated secondary antibody (GBI Labs, Bothell, WA, USA) and TSA-fluor (1:100, PerkinElmer, Santa Clara, CA, USA), after which the primary and secondary antibodies were thoroughly eliminated by heating the slides in citrate buffer (pH 6.0) for 10 min at 95 °C. In a serial fashion, each antigen was labeled with distinct fluorophores. Nuclei were subsequently visualized with DAPI (1:2000, DAKO), and the slides were coverslipped using ProLong Gold Antifade Mountant (Thermo Fisher, Waltham, MA, USA). This Opal protocol of MMP2 (1:200, Abcam, Cambridge, UK; Opal 520), MMP9 (1:200, Abcam; Opal 570), MMP11 (1:200, BGI-GBI Biotech, Beijing, China; Opal 670), Pepsin C (1:100, LifeSpan BioSciences, Seattle, WA, USA; Opal 650), E-cadherin (1:100, BD Transduction Laboratory, New York, NJ, USA; Opal 690) and S100A14 (1:100, Proteintech; Opal 620) was applied to the GC tissue microarrays. The multiple fluorophore-antibody staining on the same tissue was photographed and analyzed by PerkinElmer.

### Cell lines and cell culture

The gastric cancer cell lines BGC823, MGC803, SGC7901, AGS and N87 and gastric epithelial cells transfected with SV40 (GES-1) were cultured in Dulbecco’s modified Eagle’s medium (DMEM; Macgene, Beijing, China) supplemented with 10% fetal bovine serum (FBS; Gibco, Carlsbad, CA, USA), 100 units/ml streptomycin, and 100 units/ml penicillin. All the cell lines were maintained at 37 °C in 5% CO_2_.

### Transfection generation of stable cell lines

The specific RNAi sequences of S100A14 were designed by Invitrogen (RNAi-2:5′-GGAGUUUCUGGGAGCUGAUTT-3′ RNAi-3:5′-GAGCUGGGCUAUGGGAAAUTT-3′) and inserted into the GV248 plasmid (hU6-MCS-Ubi-EGFP; Genechem, Shanghai, China), then the plasmids and vector were transfected into AGS cells. And the cells were seeded in selection medium containing 2 *μ*g/ml puromycin (Genechem) for 2 weeks to form stable clones post-transfection (AGS-vector, AGS-Ri2 and AGS-Ri3, respectively). The pcDNA3.1-S100A14 and pcDNA3.1-vector plasmids^29^ (gifts of Professor Zhihua Liu, Chinese Academy of Medical Sciences and Peking Union Medical College, Beijing) were transfected into BGC823 cells containing 400 *μ*g/ml G418 (Gibco) to form stable clones (BGC823-S100A14 and BGC823-vector, respectively). The efficacy of transfection was identified by real-time PCR, western blotting (WB) and immunofluorescence.

### RNA extraction and RT-PCR

Total RNA was extracted from the cell lines using TRIzol (Invitrogen, Carlsbad, CA, USA) and subjected to RT-PCR and real-time PCR. The primers used are listed in Supplementary Table S1. The quantity and purity of the extracted RNA were checked using a NanoDrop spectrophotometer (Thermo Fisher, Wilmington, DE, USA). Reverse transcriptase polymerase chain reaction and PCR were performed using the Biometra PCR System (Biometra, Göttingen, Germany). Real-time PCR was performed using an Applied Biosystems apparatus (ABI, Foster, CA, USA).

### Western blot analysis

Proteins were extracted from the cells for western blot analysis. In total, 50 *μ*g protein was subjected to 12% SDS-PAGE, transferred to a PVDF membrane (Millipore, Billerica, MA, USA) and incubated with the following antibodies: S100A14 antibody (1:500, Proteintech), Pepsin C antibody (1:500, LifeSpan BioSciences), E-cadherin antibody (1:500, BD Transduction), Orai1 antibody (1:500, Abcam), STIM1 antibody (1:500, Abcam), FAK antibody (1:500, CST, Beverly, MA, USA), MMP2 antibody (1:1000, Abcam), MMP9 antibody (1:1000, Abcam) and MMP11 antibody (1:500, BGI-GBI Biotech). The membrane was then incubated with HRP-conjugated goat anti-mouse IgG (1:2000, ZSGB-BIO) or HRP-conjugated goat anti-rabbit IgG (1:2000, ZSGB-BIO). *β*-Actin antibody (1:10 000, Sigma, St. Louis, MO, USA) was used as a control. The protein level was detected by ECL (GE Healthcare, Buckinghamshire, UK). Chemiluminescence was visualized using a SmartChemi image analysis system (Sagecreation, Beijing, China).

### Immunofluorescence

The cells were grown on glass slides, washed with PBS, and fixed in 4% paraformaldehyde for 10 min. After 1 h blocking with 5% BSA (Amresco, Solon, OH, USA), the slides were incubated with anti-S100A14 (1:100, Proteintech) and anti-Vinculin (1:100, Proteintech) followed by goat anti-mouse-TRITC-conjugated IgG (1:100, ZSGB-BIO) or goat anti-rabbit-Cy5-conjugated IgG (1:100, Abcam). After the slides were washed, they were studied using a confocal fluorescence imaging microscope (LSM780, ZEISS, Göttingen, Germany).

### Cell migration and invasion assays

The migration assay was performed using Transwell plates with inserts containing 8 *μ*m pores in a polycarbonate membrane (Corning, NY, USA). The invasion assay was performed using a Matrigel Invasion Chamber (Corning). The cells (5 × 10^4^) were suspended in serum-free DMEM and seeded in triplicate in each insert. DMEM supplemented with 10% FBS was used as a chemoattractant. After 12~24 h incubation, the cells were fixed immediately with 4% formaldehyde and stained with 0.1% crystal violet. The cells that penetrated the membrane were imaged and counted.

### Mouse model

Animal experiments were carried out in strict accordance with the recommendations of the Guide for the Care and Use of Laboratory Animals of the Ethics Committee of Peking University Cancer Hospital and Institute. For the experimental pulmonary metastasis model, 5 × 10^5^ cells were suspended in 1 × Hank’s buffer (Macgene) and injected into the tail veins of female NOD/SCID mice (Vitalriver Laboratory Animals). Five mice were included in each group. The lung tumor specimens were filled with Bouin's fixative, immersed in formalin, embedded in paraffin and subjected to H&E staining and IHC staining for S100A14 6 weeks later. The lung metastasis nodes from all mice were photographed and analyzed.

### Calcium assay

The calcium assay was performed as described previously.^50^ The cells were plated onto gelatin-coated 30 mm plates 1 day before the experiments. The cells were washed with Tyrode's solution and incubated with 4 *μ*M Fluo-4 AM (Invitrogen) or Fura-4 AM (Invitrogen) for 30 min at room temperature. The cells were then washed with Tyrode's solution. To measure store-operated Ca^2+^ influx, 4 mM EGTA and 4 mM thapsigargin (TG) (Abcam) were added to deplete internal calcium stores. Ca^2+^ influx was induced by the subsequent addition of 2 mM Ca^2+^ (free) after store depletion. The change in calcium fluorescence was monitored using a confocal fluorescence imaging microscope (LSM710, ZEISS), which was performed in Professor Heping Cheng’s laboratory at the Institute of Molecular Medicine, Peking University.

### Statistical analysis

All statistical analyses were conducted using SPSS 20.0 software (Armonk, NY, USA). The data are expressed as the means±S.D. Correlations between protein expression and clinical features from patients were performed using the chi-square test (*χ*^2^-test). Kaplan–Meier survival analysis and the log-rank test were used to evaluate the prognosis of the patients. For *in vitro* and *in vivo* assays, statistically significant differences were determined by Student’s *t*-test. *P*⩽0.05 was considered statistically significant in all the cases.

## Figures and Tables

**Table 1:** The relations between the expression of S100A14 and tumor-related variables

**Variable**	**Total case**	**S100A14 expression**		***P***
		**Low**	**High**	
*Gender*				
Male	342	162	180	0.430
Female	98	42	56	

*Age*				
>60	183	75	108	0.047
⩽60	253	128	125	

*Classification*				
Intestinal	300	121	179	**<0.001**
Mix	43	22	21	
Diffuse	97	61	36	

*Differentiation*				
Poorly	224	114	110	**0.044**
Well	204	84	120	

*T stage*				
T1–T2	78	26	52	**0.014**
T3–T4	353	172	181	

*N stage*				
N0	110	39	71	**0.011**
N1–3	321	159	162	

*M stage*				
M0	393	173	220	**0.010**
M1	38	25	13	

Bold values emphasize correlation between S100A14 and the variables.
