# Supplementary material for: Calcium-binding protein S100A14 induces differentiation and suppresses metastasis in gastric cancer
Source: Cell Death Dis. 2017 Jul 20;8(7):e2938–. doi: 10.1038/cddis.2017.297 (PMC5550849; doi:10.1038/cddis.2017.297)
Supplement: Supplementary Methods and Figures legends [file cddis2017297x1.docx]

**Supplementary methods**

**Cell proliferation assays**

﻿For MTT assays, cells (2×10^3^) were seeded in triplicate in each well of 96-well culture plates, and 10 μl MTT (5 mg/ml; Genview, Florida, USA) was added to 100 μl medium every 24 h for 5 days. Absorbance was measured at 570 nm using an iMark Microplate Reader (Bio-Rad, Hercules, CA, USA). For colony formation assays, cells (1×10^3^) were seeded in triplicate into 60 mm dishes, and colonies were stained with 0.1% crystal violet after 2 weeks of culture. For soft agar assays, BGC823 cells stably transfected with S100A14 overexpression plasmid or the vector control were suspended in triplicate in DMEM containing 10% FBS with 0.3% agar (Sigma) and overlying 0.6% agar in 60 mm culture dishes. Colonies were stained with 0.2% iodonitrotetrazolium chloride (INT) (Amresco) after 4 weeks of culture.

**Tumorigenicity assay**

In total, 5×10^5^ BGC823-vector and BGC823-S100A14 cells were separately injected subcutaneously into female BALB/c nude mice (Vitalriver Laboratory Animals, Beijing, China). The mice were checked every 3 days for tumor appearance. The volume of tumor specimens from nude mice was calculated, and then the specimens were immersed in formalin, embedded in paraffin, and subjected to H&E staining and immunostaining with S100A14 four weeks later.

**Supplementary Figure legends**

**Supplementary Figure 1** The establishment of knockdown and ectopic S100A14 expression stable transfectants. (a-c) Real-time PCR (a), Western blot (b) and Immunofluorescence (c) analysis of S100A14 expression in transfected AGS cells. (d-f) Real-time PCR (d), Western blot (e) and Immunofluorescence (f) analysis of S100A14 expression in transfected BGC823 cells. Scale bar, 5 μm. The data are either representative of three similar experiments or are shown as the mean ± SD of three experiments. ** *P* ˂ 0.01; *** *P* ˂ 0.001

**Supplementary Figure 2** SKF96365, EGTA and calpeptin decreased the inhibitory effect of S100A14 on cell invasion. Cell invasion assays showed that SKF96365 (20 µM), EGTA (2 mM) and calpeptin (20 µM) decreased S100A14 knockdown-induced invasion of AGS cells and the invasion of S100A14-deficient BGC823 cells. Quantitative analysis of the cells that migrated across the matrix membrane (lower). The data are shown as the mean ± SD of three experiments. *** *P* ˂ 0.001

**Supplementary Figure 3** Functional effects of S100A14 on cell proliferation and tumorigenicity. (a and b) MTT assay (a) and colony formationassay.(b) results showed that S100A14 knockdown had no effects on cell proliferation in AGS cells. (c-e) MTT assay (c), colony formation assay (d), and soft agar assay (e) results showed that S100A14 overexpression had no effects on cell proliferation in BGC823 cells. (f) S100A14 overexpression did not affect BGC823 tumorigenicity (upper). S100A14 expression as detected by immunohistochemistry (lower). Scale bars, 50 μm. (g) The growth trend chart shows the similar sizes between BGC823-vector and BGC823-S100A14 cells in the process of tumorigenicity. The data are shown as the mean ± SD of three experiments. *P* ˃ 0.05
