# Supplementary material for: Calcium-binding protein S100A14 induces differentiation and suppresses metastasis in gastric cancer
Source: Cell Death Dis. 2017 Jul 20;8(7):e2938–. doi: 10.1038/cddis.2017.297 (PMC5550849; doi:10.1038/cddis.2017.297)
Supplement: Supplementary Table S1 [file cddis2017297x2.docx]

| Supplementary Table S1: Primer sequences for PCR | | | |
| --- | --- | --- | --- |
| Gene Name | Primer Sequence (5'→3') | Size (bp) | Tm (℃) |
| S100A14 | F: GTCGGTCAGCCAACGCAGAG | 150 | 60 |
|  | R: CAGGCCACAGTTGCTCGG |  |  |
| Orai1 | F: ATGAGCCTCAACGAGCACT | 160 | 58 |
|  | R: GTGGGTAGTCGTGGTCAG |  |  |
| STIM1 | F: CAGAGTCTGCATGACCTTCA | 125 | 58 |
|  | R: GCTTCCTGCTTAGCAAGGTT |  |  |
| MMP2 | F: AGTTTCCATTCCGCTTCCAG | 100 | 60 |
|  | R: CGGTCGTAGTCCTCAGTGGT |  |  |
| MMP9 | F: CCAACTACGACACCGACGAC | 106 | 58 |
|  | R: TGGAAGATGAATGGAAACTGG |  |  |
| MMP11 | F: CCTGCATCTGTCTGCCTTCT | 197 | 62 |
|  | R: GCTTTGGAGGATAGCAGTGC |  |  |
| β-actin | F: TTAGTTGCGTTACACCCTTTC | 150 | 56 |
|  | R: ACCTTCACCGTTCCAGTTT |  |  |
