# Supplementary material for: Calcium-binding protein S100A14 induces differentiation and suppresses metastasis in gastric cancer
Source: Cell Death Dis. 2017 Jul 20;8(7):e2938–. doi: 10.1038/cddis.2017.297 (PMC5550849; doi:10.1038/cddis.2017.297)
Supplement: Supplementary Table S2 [file cddis2017297x3.docx]

Supplementary Table S2: Analysis of S100A14 expression in gastric cancer

| Histology | Total cases | S100A14 Expression | | *P* |
| --- | --- | --- | --- | --- |
|  |  | Low (%) | High (%) |  |
| Normal tissue | 289 | 123 (42.6) | 166 (57.4) | 0.313 |
| Gastric tumor | 440 | 204 (46.4) | 236 (53.6) |  |
